# Early recognition of risk factors for adverse outcomes during hospitalization among Medicare patients: a prospective cohort study

**DOI:** 10.1186/1471-2318-13-72

**Published:** 2013-07-08

**Authors:** Jeff Borenstein, Harriet Udin Aronow, Linda Burnes Bolton, Jua Choi, Catherine Bresee, Glenn D Braunstein

**Affiliations:** 1 Applied Health Services Research, Cedars-Sinai Health System, 8700 Beverly Blvd, Los Angeles, CA 90048, USA; 2 Nursing Research, Cedars-Sinai Health System, 8700 Beverly Blvd, Los Angeles, CA 90048, USA; 3 Cedars-Sinai Health System, 8700 Beverly Blvd, Los Angeles, CA 90048, USA

**Keywords:** Frailty, Readmissions, Patient safety, Medicare, Hospitalized elderly

## Abstract

**Background:**

There is a persistently high incidence of adverse events during hospitalization among Medicare beneficiaries. Attributes of vulnerability are prevalent, readily apparent, and therefore potentially useful for recognizing those at greatest risk for hospital adverse events who may benefit most from preventive measures. We sought to identify patient characteristics associated with adverse events that are present early in a hospital stay.

**Methods:**

An interprofessional panel selected characteristics thought to confer risk of hospital adverse events and measurable within the setting of acute illness. A convenience sample of 214 Medicare beneficiaries admitted to a large, academic medical center were included in a quality improvement project to develop risk assessment protocols. The data were subsequently analyzed as a prospective cohort study to test the association of risk factors, assessed within 24 hours of hospital admission, with falls, hospital-acquired pressure ulcers (HAPU) and infections (HAI), adverse drug reactions (ADE) and 30-day readmissions.

**Results:**

Mean age = 75(±13.4) years. Risk factors with highest prevalence included >4 active comorbidities (73.8%), polypharmacy (51.7%), and anemia (48.1%). One or more adverse hospital outcomes occurred in 46 patients (21.5%); 56 patients (26.2%) were readmitted within 30 days. Cluster analysis described three adverse outcomes: 30-day readmission, and two groups of in-hospital outcomes. Distinct regression models were identified: Weight loss (OR = 3.83; 95% CI = 1.46, 10.08) and potentially inappropriate medications (OR = 3.05; 95% CI = 1.19, 7.83) were associated with falls, HAPU, procedural complications, or transfer to intensive care; cognitive impairment (OR = 2.32; 95% CI = 1.24, 4.37), anemia (OR = 1.87; 95% CI = 1.00, 3.51) and weight loss (OR = 2.89; 95% CI = 1.38, 6.07) were associated with HAI, ADE, or length of stay >7 days; hyponatremia (OR = 3.49; 95% CI = 1.30, 9.35), prior hospitalization within 30 days (OR = 2.66; 95% CI = 1.31, 5.43) and functional impairment (OR = 2.05; 95% CI = 1.02, 4.13) were associated with 30-day readmission.

**Conclusions:**

Patient characteristics recognizable within 24 hours of admission can be used to identify increased risk for adverse events and 30-day readmission.

## Background

The Institute of Medicine report on patient safety in the U.S. health care system, *To Err Is Human*, highlighted the unacceptably high incidence of adverse events during hospitalization [1]. More recently, the Office of the Inspector General reported that 13.1% of hospitalized patients with Medicare insurance experienced an adverse event that resulted in harm [2]. Clearly, opportunities to improve patient safety remain, particularly among Medicare beneficiaries.

Frailty is a term describing a state of general debility associated with decline, disability, loss of independence, susceptibility to iatrogenic complications, and poor health outcomes [3,4]. As such, frailty is a potentially useful construct for identifying those within the Medicare population who are most vulnerable to adverse events associated with hospitalization. Prompt recognition of frailty could facilitate communication, multidisciplinary care coordination, risk reduction interventions, prognostication, and appropriate treatment plan development [5,6].

Although frailty has long recognized as a clinical syndrome within the field of geriatrics, there is no universally accepted definition [7,8]. Alternative approaches to identifying frailty employ significantly differing methods, and vary in their strengths and limitations [9]. Existing models of frailty were primarily developed in outpatient cohorts, and are therefore challenging to apply to an inpatient population [10-13]. A validated and widely used measure defines frailty as a deficit in at least three of five measures of function, one of which is walking speed [10]. This functionally-based strategy can be difficult to assess in the setting of acute illness. Frailty indices that quantitate the sum of accumulated deficits across a wide range of possibilities are somewhat complex to apply in practice, as they typically require inclusion of at least 30 or more variables [14]. ‘Vulnerability’, a related construct, represents an impending risk of functional decline, and can be assessed with a simple screening tool, the Vulnerable Elders Survey-13 (VES-13) [11]. Both frailty and vulnerability describe a health state that is fragile, associated with adverse health events, and more easily recognized with a global view of wellness rather than any specific medical condition. The VES-13 requires patient self-report of function over the preceding 4 weeks, which could also be affected by factors leading to hospitalization. None of these approaches to identifying frailty, as functional deficit, a composite index, or vulnerability to impending decline, have been well-validated within a medical inpatient cohort.

We conducted a prospective cohort study in a convenience sample of Medicare patients to test the hypothesis that a set of risk factors associated with frailty and identifiable within 24 hours of hospital admission would be associated with adverse events during hospitalization.

## Methods

Patient characteristics that could be potentially associated with adverse events during hospitalization were derived by consensus by an interprofessional quality improvement (QI) workgroup. Sources for candidate variables included a search of the PubMed database using the terms “frail”, “frailty”, “vulnerable”, “vulnerability, and “fragile”, and discussions with local topic experts. A Delphi panel of physicians, nurses, and allied health care providers selected items readily measurable, identifiable on admission, thought to have a relatively high likelihood of an association with adverse health outcomes and be potentially amenable to risk reduction strategies [1,15]. Given the large number of candidate variables [8,16], we further sought to identify a subset of practical attributes that were clinically relevant to the patient population at our institution and representative of a multidisciplinary perspective. To this end, the subset of risk factors for consideration were selected over the course of three meetings in which physicians and nurses were equally represented and comprised approximately two-thirds of the 20–25 attendees. The remainder of attendees were mostly allied health care professionals, as described in Additional file 1 Following a baseline vote, rounds of discussion and anonymous re-voting continued until consensus was achieved, defined as all votes falling within one of the mode on a one to nine scale of increasing disagreement, where scores of 1–3 indicated agreement. Exploratory variables included admission from a skilled nursing facility [5], age ≥80 years [17,18], presence of a feeding tube [19], and decubitus ulcers noted on admission [20]. Additional exploratory variables include the presence of four or more active comorbid conditions, anemia, cognitive impairment, deconditioning, dehydration, a positive screen for depression, functional impairment, high burden of comorbid illness, hyponatremia, hypoalbuminemia, polypharmacy, early readmission, and recent unintentional weight loss. Definitions of these terms are provided in Additional file 1.

The candidate set of risk factors was then evaluated in a convenience sample of patients ages 35 years and older with Medicare insurance from a total of n = 653 admitted to general medical/surgical units within our institution in September 2010. Only patients who were accessible to the nurses performing the assessments and who agreed to the extra assessments and to have their chart information reviewed were included. Patients were interviewed and their charts reviewed within 24 hours of admission. Other patient characteristics were derived from a variety of sources: Nurses administered the VES-13, and assessments of functional status (Katz Assessment for Functional Status) [21], cognitive impairment (Brief Interview for Mental Status) [22] and symptoms of depression (Patient Health Questionnaire-2) [23]. Clinical pharmacists documented the use of potentially inappropriate medications prior to admission [24,25], and adverse drug events during hospitalization. The latter were identified by the occurrence of a sentinel event or “trigger” and confirmed with chart review [26]. Specific criteria used in medication review are described in Additional file 1. Patient falls, hospital-acquired pressure ulcers, and readmissions within 30 days of discharge were obtained from administrative and patient safety databases, and medical record auditing. Physicians blinded to the initial nursing assessment reviewed medical charts recorded all other clinical outcomes.

This work of the Frailty workgroup was provided administrative approval by the Cedars-Sinai Medical Center Institutional Review Board as an evidence-based (QI) project. For the purposes of the current research analyses, the data from the QI project were de-identified and used secondarily. The Institutional Review Board approved request for waiver for the need to obtain consent from participants.

### Statistical analysis

Research analyses were performed using SAS (The SAS Institutes Incorporated, Cary, NC, release 9.3). Descriptive statistics were produced for all frailty factors and adverse outcomes. (see Table 1) Associations among adverse outcome variables were assessed with Spearman rank correlations. Due to multiple low frequency events and high inter-correlation among adverse outcomes data, variable cluster analysis was performed to develop a smaller number of independent outcomes [27]. Variable clustering was performed using a linear combination of the first principal component and following a hierarchical divisive structure. The final number of clusters of adverse outcome events was determined empirically and confirmed clinically. Internal consistency of each cluster was evaluated with Cronbach’s alpha. Un-adjusted and then multivariable, adjusted, logistic regression modeling were used to determine the set of frailty factors that were predictive of the presence or absence of any one or more events within each adverse outcome cluster using the selection criteria as described by Collett [28]. The final multivariable models were assessed for goodness-of-fit by inspection of the Pearson residuals for identification of observations poorly accounted for in each model. The c-statistic (the area under the receiver-operator curve) was computed for each multivariable model to evaluate model discrimination. The cumulative effect of each frailty characteristic on adverse events was also tested by Spearman rank correlation. Data are presented as means +/− standard deviations, or counts and percentages. Data were considered statistically significant where p < .05.

**Table 1:** Study cohort demographics

**Description**	**n (%)**
Female	123 (57.9)
Age (years)	
<65	40 (18.7)
65-79	85 (39.7)
> 80	89 (41.6)
Race	
White	147 (68.7)
Black	47 (22.0)
Other	20 (9.4)
Secondary insurance type	
Commercial PPO^a^	61 (28.5)
Commercial Indemnity	41 (19.2)
Medicaid Indemnity	78 (36.4)
Other/Unknown	34 (15.9)
Discharge destination	
Home/self-care	112 (52.3)
Home health care	46 (21.5)
Skilled Nursing Facility	36 (16.8)
Hospice	6 (2.80
Expired	3 (1.4)
Other	11 (5.1)
Comorbidities (n and% of total for each)	
Myocardial infarction	22 (10.3)
Heart failure	36 (16.8)
Ischemic heart disease	21 (9.8)
COPD^b^	28 (13.1)
Peripheral vascular disease	28 (13.1)
Diabetes	66 (30.8)
Cancer	44 (20.6)
Dementia	28 (13.1)
Hepatic disease	14 (6.6)
Mild renal disease^c^	24 (11.2)
Moderate/severe renal disease^c^	72 (33.6)
Frailty characteristics (n and% of total for each)	
Admitted from a skilled nursing facility	22 (10.3)
Ages 80 years and older	89 (41.6)
Anemia	103 (48.1)
Charlson Comorbidity Index Score > =4	74 (34.6)
Cognitive impairment	76 (35.5)
Deconditioning	28 (13.1)
Decubitus ulcer	17 (7.9)
Dehydration	84 (39.3)
Depression screen positive*	75 (42.1)
Feeding tube present at admission	8 (3.7)
Four or more active comorbid conditions	158 (73.8)
Functional impairment	77 (36.0)
Hyponatremia	20 (9.3)
Malnutrition	27 (12.6)
Potentially inappropriate medications	72 (33.6)
Use of a major tranquilizer	49 (22.9)
Polypharmacy**	106 (51.7)
Early readmission:	
≥1 within the past 30 days	59 (27.6)
≥2 within the past 6 months	49 (22.9)
Recent unintentional weight loss	41 (19.2)

^a^PPO = Preferred Provider Organization; ^b^COPD = chronic obstructive pulmonary disease; ^c^Renal disease severity determined by estimated creatinine clearance: mild = 60–89 ml/min;moderate/severe = <60 ml/min or dialysis dependent. Data available from only (*)n = 178 or (**)n = 205.

## Results

### Patient characteristics

The study cohort was comprised of 214 patients admitted to our institution in September 2010. Mean patient age was 75+/− 13.4 years, and mean length of hospital stay was 5.8 +/−6.26 days. The most prevalent risk factors were four or more active comorbidities (73.8%), polypharmacy (51.7%), and anemia (48.1%) (Table 1). Among those able to complete the VES-13 (n = 161, 75.2%), nearly two-thirds (n = 106, 65.8%) met criteria for vulnerability (mean score 5.0 +/− 2.7). All frailty characteristics, with the exception of a hospitalization within 30 days prior to admission, evidence of recent weight loss, and the presence of a feeding tube on admission, were associated (p < .05) with vulnerability per the VES-13 scale (data not shown). Over half (54%) of patients were prescribed one or more potentially inappropriate medications prior to admission, of which major tranquilizers were the most common subcategory.

### Adverse outcomes of hospitalization and cluster analysis

Adverse patient outcomes and their frequencies are presented in Table 2. The incidence of patient readmissions within 30 days of hospital discharge and a length of hospital stay (LOS) 7 days or longer were 21.0% and 26.2%, respectively. In all, 41 patients (19.2%) experienced any adverse event during hospitalization, with adverse drug events being the most common (11.7%). Statistically significant intercorrelations (P < .05) among adverse outcomes were observed for all variables except hospital-acquired infections and readmissions with 30 days (data not shown).

**Table 2:** Incidence of adverse outcomes (N = 214 patients)

**Outcome**	**n (%)**
Any adverse events during hospitalization	46 (21.5)
Adverse drug events	25 (11.7)
Hospital-Acquired Infections	11 (5.1)
Transfer to intensive care	12 (5.6)
Complications of a medical procedure	12 (5.6)
Hospital-acquired pressure ulcers	2 (0.9)
Falls during hospital	5 (2.3)
Length of hospital stay 7 days and longer	56 (26.2)
Mortality during hospitalization	3 (1.4)
Readmission within 30 days of hospital discharge	45 (21.0)

Cluster analysis identified three distinct outcome categories: Readmission within 30 days post-discharge and two interrelated groups (clusters) of adverse events during hospitalization: The first cluster was comprised of falls, hospital-acquired pressure ulcers, complications of a medical procedure, and transfers to an intensive care unit (Cronbach’s alpha = 0.685). The second cluster was comprised of adverse drug events, length of stay 7 days or longer, and hospital-acquired infections (Cronbach’s alpha = 0.548). Results of the cluster analysis did not differ significantly when the population was restricted to patients age 65 and older.

### Relationship of potential characteristics of a frailty in a medicare population to adverse outcomes

Associations between individual characteristics and the three outcome categories identified by cluster analysis are displayed in Table 3. Significant associations are bolded. No single characteristic was significantly associated with all three outcomes.

**Table 3:** Unadjusted logistic regression modeling

**Description**	**Falls, HAPU^a^, PC^b^, ICU^c^ transfer**	**HAI^d^, ADE^e^, LOS^f^ > 7 days**	**Readmission**
			**within 30 days^h^**
	**[Cluster 1]**	**[Cluster 2]**	
	**OR (95% CI)**	**OR (95% CI)**	**OR (95% CI)**
≥4 active comorbid conditions^g^	0.87 (0.32, 2.38)	**2.46 (1.15, 5.24)**	1.58 (0.71, 3.53)
Admitted from a skilled nursing facility	0.40 (0.05, 3.14)	1.35 (0.54, 3.39)	1.61 (0.59, 4.44)
Ages 80 years and older	0.54 (0.14, 2.11)	2.60 (0.98, 6.91)	1.39 (0.53, 3.61)
Altered mental status	1.62 (0.44, 6.04)	0.91 (0.34, 2.46)	0.92 (0.29, 2.89)
Anemia	1.49 (0.60, 3.71)	**2.40 (1.32, 4.37)**	**2.37 (1.20, 4.69)**
Charlson Comorbidity Index Score > =4	0.56 (0.20, 1.60)	1.54 (0.84, 2.82)	1.56 (0.79, 3.06)
Cognitive impairment	**0.27 (0.08, 0.96)**	**2.31 (1.27, 4.22)**	0.78 (0.38, 1.57)
Deconditioning	0.31 (0.04, 2.39)	1.88 (0.84, 4.25)	0.86 (0.31, 4.43)
Decubitus ulcer present at admission	1.21 (0.26, 5.69)	**3.62 (1.31, 10.01)**	**3.31 (1.15, 9.47)**
Dehydration	1.18 (0.47, 2.94)	1.65 (0.91, 2.98)	1.55 (0.79, 3.01)
Delirium	1.73 (0.00, 10.53)	0.76 (0.08, 7.45)	1.24 (0.13, 12.16)
Depression screen positive*	1.00 (0.38, 2.62)	1.85 (0.95, 3.59)	1.51 (0.73, 3.11)
Feeding tube present at admission	1.33 (0.16, 11.46)	4.06 (0.94, 17.51)	1.24 (0.24, 6.36)
Functional impairment	0.53 (0.19, 1.49)	**2.46 (1.35, 4.49)**	**2.29 (1.17, 4.48)**
Hyponatremia	1.02 (0.22, 4.75)	1.60 (0.62, 4.13)	**3.52 (1.36, 9.13)**
Hypoalbuminemia	1.17 (0.32, 4.28)	**2.41 (1.06, 5.47)**	0.91 (0.32, 2.58)
Potentially inappropriate medications	**2.93 (1.17, 7.34)**	0.91 (0.49, 1.69)	1.13 (0.57, 2.27)
Use of a major tranquilizer	2.28 (0.89, 5.88)	1.15 (0.58, 2.28)	1.47 (0.70, 3.08)
Polypharmacy**	0.75 (0.28, 1.99)	1.08 (0.60, 1.95)	1.02 (0.53, 1.98)
Recent admissions:			
>1 within the past 30 days	0.41 (0.12, 1.44)	1.39 (0.74, 2.64)	2.95 (1.48, 5.87)
≥2 within the past 6 months	0.74 (0.22, 2.42)	1.64 (0.84, 3.20)	1.75 (0.84, 3.66)
Recent unintentional weight loss	**3.68 (1.43, 9.46)**	**2.98 (1.48, 6.02)**	1.02 (0.44, 2.32)

^a^HAPU = hospital-acquired pressure ulcer(s); ^b^PC = procedural complications; ^c^ICU = intensive care unit; ^e^HAI = hospital-acquired infection(s); ^f^LOS = Length of hospital stay. ^g^At least one of the conditions was required to be “uncontrolled” (not at therapeutic goal). ^h^Three patients died during admission and were excluded from the analysis of readmissions. Complete data available from only (*)n = 178 or (**)n = 205.

**Figure 1:**
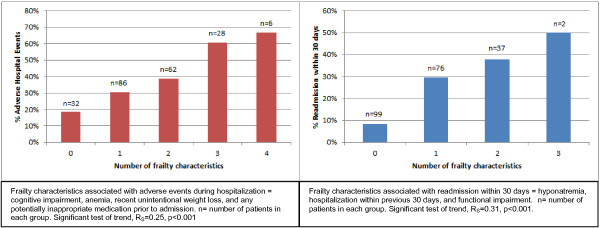
**Proportion of adverse events and number of patient frailty characteristics.** Frailty characteristics associated with adverse events during hospitalization = cognitive impairment, anemia, recent unintentional weight loss, and any potentially inappropriate medication prior to admission. n= number of patients in each group. Significant test of trend, R_S_=0.25, p<0.001. Frailty characteristics associated with readmission within 30 days = hyponatremia, hospitalization within previous 30 days, and functional impairment. n= number of patients in each group. Significant test of trend, R_S_=0.31, p<0.001.

The final adjusted regression models (Table 4) resulted in seven frailty characteristics emerging as significant independent predictors in the three logistic regressions of adverse outcome clusters and readmissions: cognitive impairment; anemia; recent unintentional weight loss; any potentially inappropriate medication; hyponatremia; hospitalization within preceding 30 days; and functional impairment. Only one variable, unintentional weight loss, was associated with both inpatient outcome clusters. Hyponatremia was an independent risk factor of readmission within 30 days, irrespective of comorbid renal disease, diabetes, or heart failure. Furthermore, we found that the incidence of adverse outcomes increased proportionally to the number of associated frailty characteristics (p < .001 for both comparisons, Figure 1).

**Table 4:** Multivariable logistic regression models for characteristics associated with adverse hospital outcomes

**Characteristics**	**Falls, HAPU^a^, PC^b^, or ICU^c^ transfer**	**HAI^d^, ADE^e^, or LOS^f^ > 7 days**	**Readmission within 30 days**
	**[Cluster 1]**	**[Cluster 2]**	
	**OR (95% CI)**	**OR (95% CI)**	**OR (95% CI)**
Anemia		1.87 (1.00, 3.51)**	
Any potentially inappropriate medication	3.05 (1.19, 7.83)**		
Cognitive impairment		2.32 (1.24, 4.37)*	
Functional impairment			2.05 (1.02, 4.13)**
Hospitalization within preceding 30 days			2.66 (1.31, 5.43)*
Hyponatremia			3.49 (1.30, 9.35)**
Recent unintentional weight loss	3.83 (1.46, 10.08)*	2.89 (1.38, 6.07)*	
c-statistic	0.718	0.686	0.713

^a^HAPU = hospital-acquired pressure ulcer(s); ^b^PC = procedural complications; ^c^ICU = intensive care unit; ^d^HAI = hospital-acquired infection(s); ^e^LOS = Length of hospital stay *p < .01, **p < .05.

## Discussion

In this prospective study of Medicare beneficiaries, characteristics readily identifiable on hospital admission were associated with an increased risk of adverse events and subsequent hospitalization within 30 days of discharge. Potential risk factors were derived from published literature on frailty, a construct developed largely in the ambulatory setting. Candidate variables were selected by a multidisciplinary panel that took into consideration practical constraints associated with acute illness, such as limitations on mobility and strength.

The goal of this research was to identify patient characteristics associated specific inpatient adverse events as a basis for subsequent targeting of risk mitigation efforts. We chose to perform the study in a cohort of Medicare patients due to their relatively high risk of adverse events during hospitalization [29]. As a consequence, the majority of patients included in the analysis (81.3%) were 65 years old and older, over half of whom (51.2%) were at least 80 years of age. The projected rapid growth of the population of older adults, and the associated rise in anticipated use of health services, makes improving the inpatient safety among the elderly an urgent priority [30,31]. Adverse medical events occur with greater frequency among the elderly, and can lead to functional decline, decreased quality of care, and increased costs [32,33]. The elderly also account for the greatest proportion of the rising number of hospital admissions, but performance on quality indicators (QIs) for common geriatric issues lags behind that of other medical conditions [34,35]. Among 349 hospital patients ages 65 years and older meeting VES-13 criteria for vulnerability, Arora et al. found that QIs for delirium and dementia were satisfied less frequently than QIs for general medical care (31.4% and 81.5%, respectively) [35]. Jencks et al. reported that over a 15-month period between 2003 and 2004, nearly one-fifth of all Medicare beneficiaries were readmitted within 30 days of hospital discharge. Such unplanned readmissions accounted for an estimated 17% of the $102.6 billion in hospital payments made by Medicare in 2004 [36].

In order to be useful for a prompt response and risk mitigation efforts, we limited our investigation to risk factors that were recognizable within 24 hours of hospital admission. Attributes commonly associated with frailty are highly prevalent among the elderly population, and therefore potentially useful for studies of risk factors for adverse events among hospitalized Medicare patients [3]. Searle et al. described a standardized process for developing frailty indices by examining the association of specific deficit and mortality in a community-dwelling cohort [37]. As noted in a position statement of the American Geriatrics Society (AGS), interdisciplinary assessment and care have been shown to improve health outcomes in the elderly in a variety of settings [38]. Combining these two concepts, we employed multidisciplinary consensus to select among a large number of potential frailty traits, and then examined associations with adverse events occurring more commonly in the elderly.

Initiated as part of a quality improvement effort at our institution, this strategy follows a practical approach to identifying vulnerability within a hospitalized population that is broadly applicable [37]. The observed clustering of outcomes minimizes the sample size required for statistical modeling. This phenomenon, together with the use of readily identifiable patient characteristics, makes these types of analyses feasible, even in resource-constrained environments. The value of such efforts, however, will depend entirely on future demonstration of their usefulness in facilitating effective risk reduction.

Our approach differed from prior work in that we used prospective data, and focused on characteristics present early in hospitalization. A recent systematic review of risk prediction models of readmission by Kasangra et al. identified only two contemporary studies that used real-time data [39]. Neither model included information available within 24 hours of admission [40,41]. We observed that health issues readily identifiable on admission – hyponatremia, functional impairment, and prior admission within 30 days – were associated with early readmission, and distinct from predictors of adverse events during hospitalization. While not typically the reason for admission to an acute care facility, these characteristics may reflect a poor state of general health. Consequently, medical management that focuses solely on the immediate causes of hospitalization may have little impact on underlying frailty, and leave patients more susceptible to poor short and long-term outcomes, such as unplanned rehospitalizations and institutionalization. It is possible that discharge planning that includes strategies to address these risk factors could help to reduce early hospital readmission.

The results of this analysis reconfirmed some characteristics that have been found to be independently associated with adverse health outcomes in other studies. Prior hospital admission within 30 days [17] and impaired functional status as measured by the Katz Scale [42] predict readmission within 30 days of discharge. Weight loss ≥10 pounds defines an increased risk of malnutrition that is associated with a higher incidence of falls [43] and longer hospital stays [44]. Similarly, inpatient falls occur more frequently in patients prescribed potentially inappropriate medications [25], and the presence of either anemia or cognitive impairment increases the likelihood of prolonged hospitalization [45]. Community-acquired hyponatremia is associated with inpatient mortality, increased length of stay, and discharge to short or long-term care facilities [46]. To our knowledge a relationship between hyponatremia and early readmissions has not been previously described in an unselected population.

This research has several limitations. We conducted a comprehensive search of the peer-reviewed literature using general terms to increase sensitivity but did not perform a systematic review, increasing the likelihood that some characteristics of a frailty may have been overlooked. Use of a convenience cohort of patients may have introduced selection bias. Not all potential risk factors were collected in every patient due to the practicality of conducting interviews in an acutely ill population, creating a potential for response bias, particularly for the VES-13 questionnaire. As patients were identified up to 24 hours following admission, the observed association of specific characteristics with the risk of adverse outcomes may have been confounded by the care patients received between admission and assessment. The association of patient characteristics and adverse events may be influenced by differences in processes of care, particularly in the structured environment of hospitals. Hospital-based risk mitigation strategies may be applied more uniformly than in ambulatory settings, yet differ substantially among institutions. Consequently, the generalizability of our findings is unclear. However, the utility of identifying at-risk populations very early in the course of hospitalization is self-evident.

The outcomes included in this analysis are subject to the influence of the actions of different health care disciplines. For example, the incidence of falls may be affected by risk recognition and mitigation by nursing, or medications prescribed by physicians [25]. In a systematic review by Cameron et al., multifactorial interventions were associated with a relative risk of falls among hospitalized patients of RR = 0.73 (95% CI 0.56 to 0.96: I^2^ = 43%) in comparison to controls [47] Similarly, incidence of hospital-acquired infections may reflect physician decisions, such as placement of a urinary catheter, or nurses’ efforts to reduce the potential for catheter-acquired urinary tract infections. The 2009 National Healthcare Quality Report of the Agency for Healthcare Quality and Research emphasized multidisciplinary teams as a key strategy for reducing HAIs [48]. Bergkvist et al. also found that teams comprised of physicians, nurses, and pharmacists reduced the use of inappropriate medications in hospitalized elderly patients [49]. Collectively, these observations support the potential benefit of an interdisciplinary approach to frailty in hospitalized patients.

Many attributes of frailty prevalent among Medicare beneficiaries can result from potentially remediable conditions, such as functional impairment, and are neither universally irreversible nor synonymous with aging [50]. Early recognition of these characteristics during hospitalization is feasible, and affords the potential to identify and address underlying health issues that may contribute to adverse events. These findings may have implications for the development of targeted hospital-based safety and quality improvement programs, and may also be relevant to post-acute care.

## Conclusions

In conclusion, among Medicare beneficiaries, characteristics identifiable within 24 hours of hospital admission are associated with adverse hospitalizations and readmission within 30 days of discharge.

## Competing interests

The authors declare that they have no competing interests.

## Authors’ contributions

JB and HUA contributed equally to this work and both had full access to all of the data used in the study. Both contributed to the conception, design and acquisition of data. Both worked with CB (Biostatistician responsible for the data analysis) to interpret the findings. JC contributed to all pharmaceutical data collection and analysis as well as to the presentation of the pharmaceutical aspects in this manuscript. Both LBB and GDB contributed to the conception and design and general oversight of data collection, analysis and the drafting of the manuscript. None of the authors have any conflict of interest, including specific financial interests and relationships and affiliations relevant to the subject matter or materials discussed in the manuscript. No funding sources have contributed to any part of this work. All authors read and approved the final manuscript.

## Pre-publication history

The pre-publication history for this paper can be accessed here:

http://www.biomedcentral.com/1471-2318/13/72/prepub

## Supplementary Material

Additional file 1 **Composition of a multidisciplinary panel.** Definitions of Exploratory Characteristics of a Vulnerable Phenotype. Potentially inappropriate medications for use in the elderly^1-7^. Click here for file
