# Supplementary material for: Early recognition of risk factors for adverse outcomes during hospitalization among Medicare patients: a prospective cohort study
Source: BMC Geriatr. 2013 Jul 8;13:72. doi: 10.1186/1471-2318-13-72 (PMC3710470; doi:10.1186/1471-2318-13-72)
Supplement: Additional file 1 — Composition of a multidisciplinary panel. Definitions of Exploratory Characteristics of a Vulnerable Phenotype. Potentially inappropriate medications for use in the elderly1-7. [file 1471-2318-13-72-S1.doc]

Appendix 1: Composition of a multidisciplinary panel

| **Discipline** | **Representation** |
| --- | --- |
| Physicians | Geriatrics, hospital medicine and intensive care, primary care, and psychiatry |
| Nursing | Geriatrics, physical and rehabilitation medicine, palliative care, advanced practice and general nursing |
| Allied Health Professionals | Clinical pharmacy, case management, social work, physical therapy, occupational therapy, clinical nutrition |
| Other | Social science |

Appendix 2: Definitions of Exploratory Characteristics of a Vulnerable Phenotype

| **Term** | **Criterion** |
| --- | --- |
| >4 active comorbid conditions | Four or more active medical issues, at least one of which was “uncontrolled” (not at therapeutic goal) 1-3 |
| Anemia | Hemoglobin <13 g/dL in men, <12 g/dL in women 4,5 |
| Cognitive impairment | Brief Interview Mental Status score ≤ 12 or assessed as confused, somnolent, or obtunded during interview 6,7 |
| Deconditioning | Limited mobility due to chronic weakness 8,9 |
| Dehydration | Blood urea nitrogen/serum creatinine > 1810-12 |
| Depression screen positive | Patient Health Questionnaire (Levenson 1992) score >2 13-15 |
| Functional impairment | Katz Scale score < 416-18 |
| High burden of comorbid illness | Charlson Comorbidity Index Score (Charlson 1987) >416,17,19,20 |
| Hyponatremia | Serum Na < 135 meq/L21,22 |
| Hypoalbuminemia | Serum albumin <3.5 g/dL 19,23-25 |
| Polypharmacy | >7 prescription medications prior to admission7,26 |
| Early readmission | >1 within the past 30 days, >2 within the past 6 months 20,27 |
| Recent unintentional weight loss | >10 lbs or other objective measure (e.g., loose-fitting clothing) within the preceding year 2,7 |

**APPENDIX 2 REFERENCES**

**1.** Marcantonio ER, McKean S, Goldfinger M, Kleefiled S, Yurkofsky M, Brennan TA. **Factors associated with unplanned hospital readmission among patients 65 years of age and older in a Medicare managed care plan**. *Am J Med.* Jul 1999;**107**(1):13–17.

**2.** Fried LP, Tangen CM, Walston J, Newman AB, Hirsch G, Gottdiener J, Seeman T, Tracy R, Kop WJ, Burke G, McBurnie MA**. Frailty in older adults: Evidence for a phenotype**. *J Gerontol a-Biol.* Mar 2001;**56**(3):M146-M156.

**3.** Allaudeen N, Vidyarthi A, Maselli J, Auerbach A. **Redefining readmission risk factors for general medicine patients**. *J Hosp Med.* Feb 2011;**6**(2):54–60.

**4.** *Assessing the iron status of populations: including literature reviews: report of a Joint World Health Organization/ Centers for Disease Control and Prevention Technical Consultation on the Assessment of Iron Status at the Population Level.* Geneva, Switzerland 6–8 April 2004.

**5.** Zakai NA, Katz R, Hirsch C, Shilpak MG, Chaves PH, Newman AB, Cushman M. **A prospective study of anemia status, hemoglobin concentration, and mortality in an elderly cohort: the Cardiovascular Health Study**. *Arch Intern Med.* Oct 24 2005;**165**(19):2214–2220.

**6.** Mehta KM, Pierluissi E, Boscardin WJ, Kirby KA, Walter LC, Chren MM, *et al***. A clinical index to stratify hospitalized older adults according to risk for new-onset disability**. *J Am Geriatr Soc.* Jul 2011;**59**(7):1206–1216.

**7.** Satish S, Winograd CH, Chavez C, Bloch DA. **Geriatric targeting criteria as predictors of survival and health care utilization**. *J Am Geriatr Soc.* Aug 1996;**44**(8):914–921.

**8.** Laniece I, Couturier P, Drame M, Gavazzi G, Lehman S, Jolly D**. Incidence and main factors associated with early unplanned hospital readmission among French medical inpatients aged 75 and over admitted through emergency units**. *Age Ageing.* Jul 2008;**37**(4):416–422.

**9.** Kleinpell RMF, Jennings K, Bonnie M. Chapter 11. Reducing Functional Decline in Hospitalized Elderly. In: Quality AfHRa, ed. *Patient Safety and Quality: An evidence-based handbook for Nurses*. Rockville, MD2008.

**10.** Inouye SK, Bogardus ST, Jr., Charpentier PA, Leo-Summers L, Acampora D, Holford TR, Cooney LM. **A multicomponent intervention to prevent delirium in hospitalized older patients**. *N Engl J Med*. Mar 4 1999;**340**(9):669–676.

**11.** Inouye SK, Viscoli CM, Horwitz RI, Hurst LD, Tinetti ME. **A predictive model for delirium in hospitalized elderly medical patients based on admission characteristics**. *Ann Intern Med.* Sep 15 1993;**119**(6):474–481.

**12.** Podrazik PM, Whelan CT. Acute hospital care for the elderly patient: its impact on clinical and hospital systems of care. *Med Clin North Am.* Mar 2008;92(2):387–406, ix.

**13.** Arroll B, Goodyear-Smith F, Crengle S, Gunn J, Kerse N, Fishman T,Falloon K, Hatcher S. **Validation of PHQ-2 and PHQ-9 to screen for major depression in the primary care population**. *Ann Fam Med.* Jul-Aug 2010;**8**(4):348–353.

**14.** Richardson TM, He H, Podgorski C, Tu X, Conwell Y. **Screening depression aging services clients**. *Am J Geriatr Psychiatry.* Dec 2010;**18**(12):1116–1123.

**15.** Mitchell SE, Paasche-Orlow MK, Forsythe SR, Chetty VK, O’Donnell JK, Greenwald JL,Culpepper L, Jack BW. **Post-discharge hospital utilization among adult medical inpatients with depressive symptoms**. *J Hosp Med.* Sep 2010;**5**(7):378–384.

**16.** Inouye SK, Zhang Y, Jones RN, Kiely DK, Yang F, Marcantonio ER**. Risk factors for delirium at discharge: development and validation of a predictive model***. Arch Intern Med.* Jul 9 2007;**167**(13):1406–1413.

**17.** Drame M, Novella JL, Lang PO, Somme D, Jovenin N, Laniece I, Courtuner P, Heitz D, Gauvain JB, Voisin T, DeWazieres B, Gonthier R, Ankri J, Jeandel C, Saint-Jean O, Blanchard F, Jolly D. **Derivation and validation of a mortality-risk index from a cohort of frail elderly patients hospitalised in medical wards via emergencies: the SAFES study**. *Eur J Epidemiol.* 2008;**23**(12):783–791.

**18.** Wong CL, Holroyd-Leduc J, Simel DL, Straus SE. **Does this patient have delirium?: value of bedside instruments**. *JAMA*. Aug 18 2010;**304**(7):779–786.

**19.** Rozzini R, Sabatini T, Cassinadri A, Boffelli S, Ferri M, Barbisoni P, Frisani GB. **Relationship between functional loss before hospital admission and mortality in elderly persons with medical illness**. *J Gerontol a-Biol*. Sep 2005;**60**(9):1180–1183.

**20.** van Walraven C, Dhalla IA, Bell C, Etchells E, Stiell IG, Zarnke K, Austin PC, Forster AJ. **Derivation and validation of an index to predict early death or unplanned readmission after discharge from hospital to the community**. *CMAJ*. Apr 6 2010;**182**(6):551–557.

**21.** Callahan MA, Do HT, Caplan DW, Yoon-Flannery K. **Economic impact of hyponatremia in hospitalized patients: a retrospective cohort study**. Postgrad Med*.* Mar 2009;**121(**2):186–191.

**22.** Wald R, Jaber BL, Price LL, Upadhyay A, Madias NE. **Impact of hospital-associated hyponatremia on selected outcomes**. *Arch Intern Med.* Feb 8 2010;**170**(3):294–302.

**23.** Sullivan DH. **Risk factors for early hospital readmission in a select population of geriatric rehabilitation patients: the significance of nutritional status**. *J Am Geriatr Soc.* Aug 1992;**40**(8):792–798.

**24.** Jones DM, Song X, Rockwood K. **Operationalizing a frailty index from a standardized comprehensive geriatric assessment**. *J Am Geriatr Soc.* Nov 2004;**52**(11):1929–1933.

**25.** Kagansky N, Berner Y, Koren-Morag N, Perelman L, Knobler H, Levy S. **Poor nutritional habits are predictors of poor outcome in very old hospitalized patients**. *Am J Clin Nutr.* Oct 2005;**82**(4):784–791; quiz 913–784.

**26.** Huang ES, Karter AJ, Danielson KK, Warton EM, Ahmed AT. **The association between the number of prescription medications and incident falls in a multi-ethnic population of adult type-2 diabetes patients: the diabetes and aging study**. *J Gen Intern Med.* Feb 2010;**25**(2):141–146.

**27.** Smith DM, Giobbie-Hurder A, Weinberger M, Oddone EZ, Henderson WG, Asch DA, Ashton CM, Feussner R, Ginier P, Huey JM, Loo L, Mengel CE. **Predicting non-elective hospital readmissions: a multi-site study**. Department of Veterans Affairs Cooperative Study Group on Primary Care and Readmissions. *J Clin Epidemiol.* Nov 2000;**53**(11):1113–1118.

Appendix 3: Potentially inappropriate medications for use in the elderly1-7

| **Medications** | **Concerns** |
| --- | --- |
| Major Tranquilizers* | Highly addictive, Increased risk of falls/fractures, longer half-lives in elderly |
| Typical and Atypical antipsychotics | Increased central nervous system and extrapyramidal adverse effects. Increased mortality if treated for dementia related psychosis. |
| Analgesics | Increased confusion, hallucinations, Increased risk of gastrointestinal bleed, Increased orthostatic hypertension |
| Anticoagulants, Salicylates | Increased interactions with medications/diet |
| Antidepressants | Anticholinergic and sedative properties, increased seizure risk |
| Antiarrthymics | QT interval prolongation risk, toxicity if decreased renal function, Increased risk of heart failure, anticholinergic |
| Oral diabetic medications | Hypoglycemia Risk |
| Gastrointestinal Drugs &  First Generation Antihistamines | Potent anticholinergic effects, exacerbate bowel dysfunction, lead to confusion, dependence, antidopaminergic effect, aspiration risk |
| Hormones | Carcinogenic potential; decreased cardioprotective effect |
| Muscle Relaxants | Anticholinergic symptoms; central nervous system depression |
| Respiratory drugs | Pseudoephedrine (hypertension or bladder outflow obstruction); Theophylline (insomnia) |
| Stimulant drugs | Central nervous system stimulant adverse effects |
| Urinary drugs | Nitrofurantoin (contraindicated creatinine clearance <60 mL/sec); Tolteradine (in bladder outflow obstruction) |
| Miscellaneous | Ferrous sulfate >325 mg/day increase constipation, sleep aids increase risk of falls/fractures, acetaminophen >2 g/day |
| **Factors prompting medical chart audit for adverse drug events8** | |
| **Trigger** | **Concerns** |
| Vitamin K use | Over-anticoagulation with warfarin |
| Flumazenil use | Oversedation with benzodiazepine |
| Naloxone use | Oversedation with narcotic |
| Sodium polystyrene use | Use in induced hyperkalemia or used in renal failure |
| Partial Thromboplastin time (PTT) >118 seconds | Over-anticoagulation with heparin |
| International Normalized Ratio (INR) >6 | Over-anticoagulation with warfarin |
| Serum glucose <70 mg/dL: hypoglycemia related to insulin use | 1.Blood sugar <70 mg/dL in pt receiving insulin.  2.Blood sugar <40 mg/dL in pt receiving insulin. |
| Rising serum creatinine related to drug use | Increased creatinine in patients receiving nephrotoxic drugs (+/- 5 days from time drug given) - increase 0.5 mg/dL for creatinine <2 mg/dL or 30% increase for baseline creatinine >2 mg/dL. |
| Clostridium difficile positive stool | Clostridium difficile positive after receiving antibiotics for more than 48 hours |
| Toxic levels of antibiotics | 1. Vancomycin trough >20 mcg/mL (actual level to be noted)  2. Aminoglycoside supratherapeutic level:  a. Gentamicin or tobramycin levels peak >10 μg/mL  b. Amikacin levels peak >30 μg/mL, trough >10 μg/mL |
| Digoxin level >2 ng/mL | Toxic digoxin level |
| Lidocaine level >5 ng/mL | Toxic lidocaine level |
| Theophylline level >20 μg/mL | Toxic levels of drug |

* Includes typical and atypical psychotropics, barbiturates, and benzodiazepines.

**APPENDIX 3 REFERENCES**

**1.** Fick DM, Cooper JW, Wade WE, Waller JL, Maclean JR, Beers MH. **Updating the Beers criteria for potentially inappropriate medication use in older adults**: **results of a US consensus panel of experts**. *Arch Intern Med.* Dec 8–22 2003;**163**(22):2716–2724.

**2.** McLeod PJ, Huang AR, Tamblyn RM, Gayton DC**. Defining inappropriate practices in prescribing for elderly people: a national consensus panel**. *CMAJ.* Feb 1 1997;**156**(3):385–391.

**3.** Gill SS, Bronskill SE, Normand SL, Anderson GM, Sykora K, Lam K, Bell CM, Lee PE, Fischer HD, Herrmann N, Gurwitz JH, Rochon PA. **Antipsychotic drug use and mortality in older adults with dementia**. *Ann Intern Med.* Jun 5 2007;**146**(11):775–786.

**4.** Schneeweiss S, Setoguchi S, Brookhart A, Dormuth C, Wang PS. **Risk of death associated with the use of conventional versus atypical antipsychotic drugs among elderly patients***. CMAJ.* Feb 27 2007;**176**(5):627–632.

**5.** Wang PS, Schneeweiss S, Avorn J, Fischer MA, Mogun H, Solomon DH, et al. **Risk of death in elderly users of conventional vs. atypical antipsychotic medications**. *N Engl J Med*. Dec 1 2005;**353**(22):2335–2341.

**6.** Shrank WH, Polinski JM, Avorn J. **Quality indicators for medication use in vulnerable elders**. *J Am Geriatr Soc.* Oct 2007;**55** Suppl 2:S373-382.

**7.** Zhang Y, Baicker K, Newhouse JP. **Geographic variation in the quality of prescribing***. N Engl J Med.* Nov 18 2010;363(21):1985–1988. Supplementary Appendix 1–5.

**8.** Rozich JD, Haraden CR, Resar RK. **Adverse drug event trigger tool: a practical methodology for measuring medication related harm**. *Qual Saf Health Care.* Jun 2003;**12**(3):194–200.
